# Supramolecular solvents: a review of a modern innovation in liquid-phase microextraction technique

**DOI:** 10.3906/kim-2110-15

**Published:** 2021-11-29

**Authors:** Muhammad Saqaf JAGIRANI, Mustafa SOYLAK

**Affiliations:** 1 Faculty of Sciences, Department of Chemistry, Erciyes University, Kayseri, Turkey; 2 National Center of Excellence in Analytical Chemistry, University of Sindh, Sindh, Pakistan; 3 Technology Research and Application Center (TAUM), Erciyes University, Kayseri, Turkey; 4 Turkish Academy of Sciences (TUBA), Ankara, Turkey

**Keywords:** Supramolecular based solvents, microextraction, liquid-phase microextraction, applications

## Abstract

Supramolecular solvents (SUPRASs) have rapidly gained more attention as a potential substitute to organic solvents in the sample preparation and preconcentration process. The essential properties of SUPRAS solvents (e.g., multiple binding sites, different polarity microenvironments, the opportunity to tailor their properties, etc.) these qualities offer numerous opportunities to advance innovative sample preparation and pretreatment platforms compared to the traditional solvents. Among these qualities, certain importance is placed on theoretical and practical knowledge. That has assisted in making significant developments in SUPRAS that advance our understanding of the processes behind SUPRA’S formation. The SUPRA–solute interactions that drive extractions are explored in this context to develop knowledge-based extraction techniques. This review mainly focused on the significant application of supramolecular-based solvents (SUPRASs) in microextraction techniques. SUPRASs-based liquid-phase microextraction (LPME) is an excellent tool for extracting, simple preparation, and preconcentration from complex environmental samples. SUPRASs-LPME has a wide range of applications for analyzing food, environmental samples, pharmaceuticals, and biological samples.

## 1. Introduction

Supramolecular chemistry describes the design and structure of complex super-molecules with the smaller building blocks that hold together through the different noncovalent interlinkage [ 1]. Usually, this interlinkage is weaker than the covalent bonds and contains dipole-dipole interactions, p-p interactions, Van-der-Waals forces, hydrogen bonding, metal-ligand interactions [2]. In supramolecular chemistry, self-assembly describes the route of relatively smaller/simpler subunits corresponding to the functionalities that spontaneously interact to form highly complex supramolecular structures. Different examples acquire from nature, such as enzymes, proteins, metalloproteins, etc. DNA is one of the common examples with a double helix structure. DNA exhibits the best arrangement between different areas such as organic, macromolecular, covalent, and supramolecular chemistry that signifies the reversibility. It is an essential route for self-assembly that allows the supramolecular systems that adapt to local changes. Supramolecules (SUPRASs) provide an ideal context for the design of molecules that have interactive properties. The SUPRASs have potential specific interactions that offer infinite opportunities to manufacture different noncovalent SUPRAS structures with exclusive properties. Due to its unique properties, SUPRAS have numerous application such as luminescent materials[3] sensors [4], light-emitting devices [5], biological, gels, and materials chemistry [6, 7 ]. Cell imaging probes’ [ 8, 9 ] supramolecular chemistry has delayed wide applications in many areas having multidisciplinary associations with physical, chemical, and biological sciences, etc. [ 10]. Supramolecular chemistry is a new field of chemistry. Firstly, it was discovered in 1987 by Lehn, Pedersen, and Cram. Due to this historical achievement, these scientists were awarded the chemistry Nobel prize and designed cavitands such as crown ethers and cryptands [11]. SUPRAS plays a vital role in the preparation of complex macromolecules, such as multimetallic helicates [12], rotaxanes [13], coordination polymers, metal-organic frameworks, clusters, etc. [14]. Those studies have helped to design and preparation of complex synthetic molecular machines. Due to this significant achievement, researchers were awarded the Nobel Prize in Chemistry (2016) by Sauvage, Stoddart, and Feringa [15–24]. Many other achievements regarding the field of SUPRAs, including Leigh, have also made a significant contribution to the development of very complex interlock arrangement highlighting the importance of SUPRAS interactions and the advancement in the structural complex molecules. Over the decade, significant contributions of SUPRAS in self-assembly have assisted in understanding the ideologies behind the intermolecular interfaces and, hence, helped develop new target and functional materials. This review aims to focus on the discoveries made within the extent of supramolecular chemistry. The properties of SUPRAS formed depend on the self-assembly and structure at the molecular level and the environmental conditions. These characteristics play a significant role in the materials’ performance, behavior, and applications [17]. SUPRAS is a water immiscible liquids that is produced by the consecutive self-assembly of the amphiphilic species at two forms nano and molecular [25,26 ]. First, amphiphilic substances have been self-assembled under the critical concentrations, producing nanostructures (i.e. aqueous vesicles and reverse micelles). The self-assembly process occurs under the optimization of different parameters such as pH value, temperature, electrolyte other materials (nonsolvent) for the surfactant aggregates and is distinct from the bulk quantity of solution as a less volume surfactant rich phases SUPRAS solvents. Watanabe et al. firstly reported SUPRAS molecules were used to extract targeted analytes in 1978 [ 27]. For past years researchers have focused on using non-ionic-based SUPRAS for the targeted extraction of hydrophobic compounds from the aqueous environment [28–31]. Currently, the field of SUPRASs has expanded up to anionic [32], zwitterionic [33], and cationic [34] reverse micelles, aqueous micelles [35], and vesicles [36]. These solvents have significant scope in the field of extraction and also the polarity range [35]. Different samples have been analyzed, such as sludge [37], soil and sediment [38], biological fluids [39], food, etc. [39, 40 ]. SUPRAS has unique properties in the extraction field, which originate from the unique arrangement of the supramolecular associations. Thus, they have excellent polarity with different types of bindings could be recognized with the solutes. Also, high concentration of surfactant have been used in the SUPRASs around the 0.7–1 mg L^−1^ for the preparation of the micelle and vesicle-based SUPRA solvents, it permits excellent recovery with low time consumption, and with low limit of detection value without the need to vaporize the extracts [35].

### 1.1. Synthesis of SUPRASs

**Figure 1:**
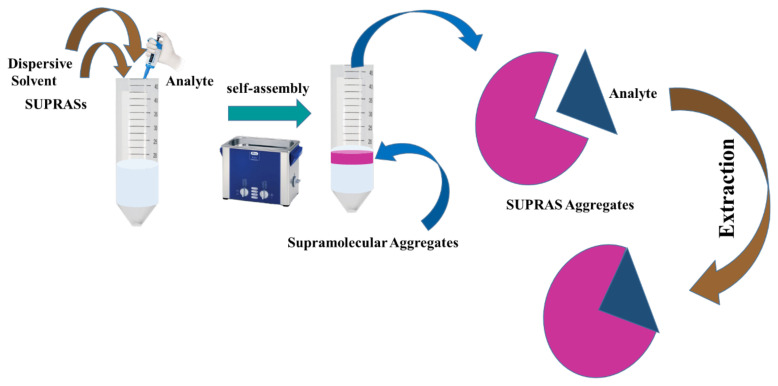
General Preparation method of SUPRAS.

SUPRASs are formed by well-defined spontaneous and sequential self-assembly and coacervation methods. Above a threshold aggregation concentration, a homogeneous solution of amphiphiles creates a colloidal solution of tri-dimensional aggregates, predominantly aqueous (36 nm) and reverse (48 nm) micelles or vesicles (30–500 nm) (ca). Environmental circumstances are changed to produce coacervation. Through this phenomenon, larger aggregates are triggered in the colloidal solution, which causes the spontaneous development of oil droplets linked and having firms of distinct droplets. Such firms’ whole thickness is altered from the solution they designed, which assists their defeating and phase separation (SUPRAS). The general preparation process of SUPRASs by using the nanostructured liquids formed in colloidal suspension solution of amphiphiles by phenomena of self-assemblage and coacervation [ 41]. The available method for their preparation contains two steps. First, the amphiphile’s aqueous or organic colloidal suspensions are ordered above the substantial aggregation concentration (cac). This suspension comprises supramolecular aggregates, characteristically aqueous or reverse vesicles or micelles. In the second step, the activity of a coacervation-inducing substance changes the ambient parameters of the colloidal suspensions, such as pH value, salts, temperature, and solvents for the amphiphile to increase the supramolecular size. The development of aggregates causes the spontaneous construction of oily droplets that associate with the clusters of distinct droplets. The conglomerates’ density differs from the prepared solution, making them flocculate or settle as new SUPRASs. The SUPRASs are colloid-rich phase, stabile with the large quantity of solution covering the amphiphile at the cac. [42]. Due to the colloid-rich phase, the SUPRASs possess more interest from the scientific community. Figure 1 shows the general synthesis process of SUPRASs

Increasing the particles’ size and making up the colloid suspensions is crucial to prepare a colloid-rich phase and coacervate. Solvophobicity induces accumulation for typical amphiphiles, while conducting the activity between the head groups is the primary factor of a stop [1]. Therefore, the repulsions of the micelles, the vesicles, etc. in colloidal suspension between head groups must be decreased in order to create coacervates. Two main pathways exist for aggregate formation that depend on the nature of the head group of the amphiphile, maybe the ionic or neutral character. The ionic networks are efficiently decreasing repulsion between groups in the charge neutralization process by adding inorganic or organic salts or amphiphilic counterions [43].

### 1.2. Interactions in SUPRASs

The extraction process has been carried out by understanding the SUPRAS–solute interactions. The solute-solvent interactions of targeted analytes have developed a SUPRA-based efficient extraction method. In this regard, significant research has been done in the previous two decades on the interactions that drive SUPRAS-based extractions. SUPRAS are primarily composed of amphiphile and water. They may also contain coacervation such as chemicals (e.g., organic or inorganic salts [36], organic solvents [44], and other components). Amphiphilic molecules have a hydrophilic and hydrophobic moiety that self-assembled and coordinated the aggregates in the SUPRAS, offering multiple polarity microenvironments. Implies can extract solutes with a wide range of polarity. As a result, they have the potential to be effective instruments for building complete sample treatment platforms before chromatography-mass spectrometry (both low and high resolution). Because the SUPRAS interactions can be fine-tuned by simply altering the amphiphiles, abundant in nature and synthetic chemistry, it’s simple to assume that SUPRAS can be tailored to meet specific needs. SUPRAS’ hydrophobic microenvironment works well as an extractant for hydrophobic substances. For solubilization principally uses dispersion and dipole-dipole generated interactions. The octanol-water distribution constants are a valuable guide to anticipate their extraction behavior since extraction efficiency rises as the hydrophobicity of the solute increases. Among the polar parts of amphiphiles, the SUPRAS-based elimination process carboxylic acids, polyethylene oxides, sulfates, sulfonates, ammonium, and pyridinium carboxylates ions. Different interactions have been reported during the extraction process using polar solutes, such as hydrogen bonding, ionic, π–cation, and π–π dipole-dipole interactions. Due to the high energy of ionic integrations, the elimination of ionic compounds with opposing charge amphiphiles is a highly efficient option [45].

#### 1.2.1. Hydrogen bonds

Hydrogen bond (H-bond) is excellent noncovalent interaction to prepare SUPRAS architectures. Due to the ideal characteristic, the H-bond is highly selective. A directional H-bond is formed when the donor with available in the acidic hydrogen atom interacts with an acceptor carrying offers non-bonding interaction. The strength mainly relies on the solvent, number, and G-bonding sequence of donor and accepter. High association constants are needed in order to create a large number of desirable H-bonded assemblies. However, weak hydrogen bond interactions produce nanosized assemblies with extra supramolecular interactions in many instances [46].

#### 1.2.2. Ionic, π–cation interactions

An active study area applies reversible interfaces between the ions and aromatic compounds to direction binding or self-assembly. This particular concern on aromatic interaction areas to progress in the research activity in these areas. Specially anionπ/weak-σ, cation π, and different secondary interactions between the leading group of cations and aromatic compounds ring that comprise a numerous ions π interactions that are used in the construction of supramolecular chemistry [47].

### 1.3. Characterization techniques used for the SUPRAS analysis

#### 1.3.1. Nuclear magnetic resonance (NMR) spectroscopy

NMR spectroscopic study of SUPRAS arises from its novel capacity to analyze the environment of the different atomic nuclei, regarding the structure and subtleties of the fashioned networks. NMR spectra provided information about the construction of the components, the resultant aggregates, and the areas participating in the interactions, which plays vital roles in the stability of the active networks. Compared to other techniques, the NMR technique is an effective characterization technique used to characterize SUPRAS. SUPRASs are indistinguishable from extensive memory, allowing nuclei to integrate different environments through chemical interactions or molecular motion. Thus, NMR is a powerful tool for examining the SUPRAS on the molecular level, and it is appropriate to provide a dynamic structure of SUPRAS [48, 49 ]. 1H NMR spectroscopy Proton NMR (1H NMR) in order to examine the interaction and the construction of molecules. The chemical shift can be changes associated with the preparation of SUPRAS that are driven by the noncovalent interactions. Fang and co-workers developed four new cholesterol-based ferrocene derivatives related to the different diamino units [50].

#### 1.3.2. Infrared (IR) spectroscopy

IR spectroscopy is a characterization technique that is widely applied for dynamics measurements, quality control, and monitoring applications. IR has also been used to analyze SUPRAS to study the functionalization and self-assembly process. IR spectroscopy gave information about hydrogen bonding and played a significant role in the SUPRAS aggregation process in the water [51, 52].

#### 1.3.3. Ultraviolet-visible spectroscopy (UV/Vis)

UV/Vis states to absorption spectroscopy. Molecules having non-bonding electrons (n-electrons) or p-electrons can absorb the energy in the form of UV or Vis light to excite the electrons to the higher antibonding molecular orbitals. The electrons can be quickly excited from the lower the energy gap among the HOMO and LUMO, the longer the wavelength of light they absorb. UV/Vis is a sample analytical technique used routinely to analyze different analytes such as biological macromolecules and highly conjugated organic compounds and 126. UV/Vis spectroscopy is also used to characterize SUPRAS because it can catch the changes in the hydrophobicity of the surrounds of a specific group that identifies the non-covalent interactions [ 53, 54 ].

## 2. SUPRASs-based LPME

The sample preparation and pre-concentration directly affect the precision, accuracy, and limit of quantification and are often the rate-determining step of the analysis process. Although the importance of sample preparation and preconcentration is often overlooked, it is a key step in the analytical process. Nowadays, the researchers focus on easy, fast, environmentally friendly, and economical friendly methods for the sample preparation. At present, the development of green, environmentally friendly, economically beneficial, and miniaturized techniques has become a key aim of research in the sample preparation process [ 55, 56 ]. Several analytical methods have been developed for the sample preparation and pre-concentration from the complex metric, such as solid-phase extraction (SPE) [ 57, 58 ], cloud point extraction [ 59, 60 ], magnetic solid-phase extraction [ 61]. The microextraction method is the best candidate to fill full the green chemistry requirements. Microextraction is a new green approach. A negligible amount of organic solvent is used for the extraction and preconcentration of the sample before analysis [62–70]. Microextraction has different modes such as vortex-assisted liquid-liquid microextraction [71, 72 ], solid-phase microextraction (SPME) [ 73–86] and liquid-phase microextraction (LPME) [75, 77, 79, 87–93 ], dispersive liquid-liquid microextraction (DLLME) [ 75, 94 ], cloud point extraction, (CPE) [ 95–99], single-drop microextraction (SDME) [100], ionic liquid-based dispersive liquid-liquid microextraction (IL-DLLME) [101, 102 ] dispersive liquid-liquid microextraction based on solidification of floating organic drop (DLLME-SFO) [103]. However, the recent trends involve the miniaturization of the conventional liquid-liquid extraction principle. The effective approach behind these is a great minimize in the volume ratio of acceptor to donor phase. Jeannot and Cantwell [104] and Liu and Dasgupta [105] presented the first research paper in 1996 on the liquid-phase microextraction. Jager and Andrews [106] and Later He and Lee [107] to share their contribution to this development. Improving accurate, precise, and ultra-sensitive analytical techniques associated with celerity and simplicity is still a difficult task to assume. Different parameters must be studied and optimized during the development of methods, and many difficulties can be found, especially in the sample preparation and pretreatment. LPME has gained more attention from researchers due to its easy extraction process. To improve the extraction efficiency and reduce the time-consuming steps, the researchers focus on the LPME technique to eliminate targeted analytes from the complex sample matrix. The LPME is cheaper, greener, fast, economically beneficial, highly selective, and sensitive sample preparation and pre-concentration methods. In LPME, very low amount of toxic organic solvents was used during the extraction process [108–111]. Currently, the LPME pays more attention to green solvents like ionic liquids [112] and SUPRASs to minimize the use of toxic organic solvents during the extraction of targeted analytes. Due to its unique properties, SUPRASs have been used in the extraction field. The SUPRASs are cheaper and greener solvent, non-volatile and non-flammable [113–115]. In recent years SUPRASs has been used as an extraction solvent in the LPME for the extraction of several targeted analytes such as benzimidazolic fungicides in aqueous media [116], endocrine disruptors in sediment [117], mecoprop and dichlorprop in soil [118], tetracyclines in food samples [119, 120 ] and Sudan dyes in foodstuffs [ 114, 116, 121–126 ]. SUPRASs are called new generation extraction solvents [ 41, 127–129 ]. SUPRASs are micro and nanostructured liquids produced in the colloidal solutions of the spontaneous amphiphilic compounds self-assembled and undergo the coacervation (rich in macromolecules) phenomena. This regular construction process of SUPRASs offers an outstanding extraction process for the selective and sensitive extraction of analytes. Therefore, at present, they have been applied for the extraction of radioactive elements, heavy metals, dyes, pesticides, antioxidants, etc. [ 121, 130–132 ].

**Figure 2:**
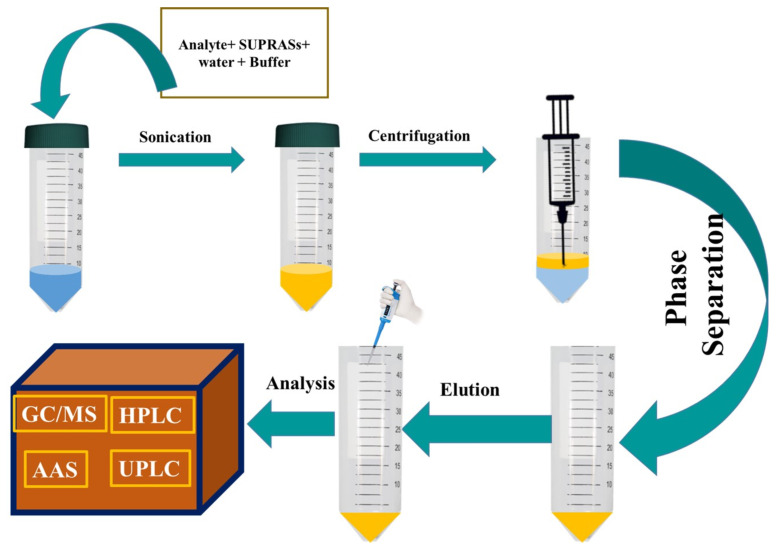
Schematic representation of SUPRASs based extraction of targeted analytes.

Due to the outstanding properties of SUPRASs for the effective solubilization of solutes in an excellent polarity range, they have found wide applications in the extraction and pre-treatment of samples [ 34, 133, 134 ]. The extraction process in the liquids samples is generally carried out using in situ because of its reversible nature and straightforward process [ 135]. Moreover, the coacervate is highly viscous. Thus, it needs to be diluted with an appropriate solvent before proceeding to any analysis. The diluted coacervate would reduce the extraction efficacy. Firstly, SUPRAS is prepared before it is applied for the extraction of targeted analytes. The two-step process is operationally more suitable because a high volume of the SUPRAS can be designed. It is typically enough to extract 10–30 samples, and it has excellent extraction efficiency because only a minimal amount of SUPRAS is needed [117, 136, 137 ]. Figure 2 shows the schematic representation of SUPRASs based extraction of targeted analytes.

## 3. Applications of SUPRASs in microextraction field

Alkanol-based preparation of SUPRASs, are generally long-chain alcohols used as amphiphiles in the aqueous media (water-miscible solvents), such as tetrahydrofuran. spontaneously the alkanols from reverse micelles through the self-assembly method resulting in the construction of SUPRASs [ 114, 138, 139 ] due to the self-assembly at the censorious accumulation of the amphiphilic molecules [ 139]. The accumulation is not produced at a concentration below the censorious accumulation, and subsequently, inadequate and ineffective extraction arises. On the other side, high amphiphiles concentration has been used to make the equal ratio of water in the ternary assortment (amphiphiles/THF/water) partially soluble and less efficient [138]. The aqueous cavities are designed in the SUPRASs when the hydroxyl group of alkanols (act as polar) of amphiphilic molecules surround the aqueous media with the non-polar hydrocarbons chain was interacting with the THF [114]. THF plays dual characteristics throughout the formation of SUPRASs. It causes the distribution of the amphiphiles in the sample and supports their self-assembly [138]. The alkanol-based SUPRASs are highly efficient for extracting organic pollutants, including food, pesticides, and environmental samples [140, 141 ]. Deng et al. [ 139] reported alkanol-based SUPRASs used for the microextraction of fluorine-containing pesticides from an aqueous environment. Thorough the separation of SUPRAS from the bulk quantity of solution was further assisted by centrifugation. The enriched phase of undecanol was isolated and diluted with the acetonitrile before the LC-MS analysis. A relatively good extraction percentage was obtained about 81.3% to 105.9%. ALOthman et al. [138] proposed alkanol-based SUPRAS using THF/heptanol and water for the selective microextraction of carbaryl pesticides from the fruit, vegetables, and water. The proposed method has been successfully applied to extract carbaryl pesticides, and the extracted sample has been diluted with ethanol before the analysis by the UPLC-MS/MS. The proposed method was environmentally friendly, using a very small amount of organic solvent during the pre-treatment process. Good extraction recoveries have been obtained up to 90% and 102%. Peyrovi and Hadjmohammadi [140] proposed undecanol-based SUPRASs for the microextraction of organophosphate pesticides from the orange juice and aqueous environment. The ternary mixture of undecanol, THF, and water. This proposed method has been achieved good percentage recoveries up to 94%. Also, a different scientist has used decanol-based SUPRASs for the sample preparation and pre-concentration of pesticides from the environmental and food samples [114, 141, 142 ]. The fatty acids-based SUPRAS are constructed using short-chain fatty acids as amphiphiles in the aqueous media containing miscible water such as THF [ 143, 144 ]. The fatty acids contain the carboxyl group (-COOH). It acts as hydrophilic while the hydrocarbon chain is lipophilic. Thus, fatty acids act as hydrophilic and hydrophobic when mixed with THF and water, and spontaneously they form reverse micelle through the self-assembly procedure. The resultant product is called SUPRASs [ 130, 145 ]. The self-assembly and aggregation occur at the hydrophilic and hydrophobic (amphiphilic) fatty acid molecule [ 139]. During the preparation of SUPRASs, a ternary mixture of fatty acids THF and water has been used because the THF has two fundamental roles during the construction of fatty acids-based SUPRASs. In the case of distribution of the fatty acids, amphiphilic molecules in the solution helps their self-assembly. Different researchers have successfully used medium-chain fatty acid-based SUPRASs for the effective pretreatment of toxic pesticides from the environmental and food samples [130, 144, 145 ]. Gorji et al. [143] proposed a decanoic acid-based SUPRASs based method for the microextraction and pre-concentration of different types of four organophosphate ( such as diazinon, phosalone, ethion, and chlorpyrifos) and an acaricide (hexythiazox) and isothiazolidine in the rice and vegetables samples. Prior to HPLC-UV analysis, the pesticides samples have been successfully extracted and pretreatment using suitable organic solvent in the small quantity. The proposed method obtained good extraction percentage from 102%–178%. Amir et al. [68] also used decanoic acid-for the preparation of SUPRASs for the microextraction and preconcentration of herbicide (phenylurea) (linuron, monuron and isoproturon) from the aqueous environment and rice samples after the sample extraction and pretreatment HPLC analysis has been carried out. The proposed method has achieve good percentage recovery up to from 80% to 90%. Fatemeh Rezaei et al. reported highly efficient and facile decanoic acid-based SUPRASs for the microextraction of benzodiazepine drugs from the aqueous samples. To prepare decanoic acid-based SUPRASs using a ternary mixture of decanoic acid water and tetrabutylammonium Bu_4_N^+^, the targeted analyte has been successfully extracted and preconcentrated before HPLC analysis. The proposed method achieves a good percentage recovery from 90.0%–98.8%. Table 1 represents the different applications of SUPRASs for the extraction of pesticides and herbicides.

### 3.1. SUPRASs based extraction of metals from different environment samples

Metal ions contamination is one of the biggest problems for human beings due to its adverse effects on the environment [ 151–155]. Due to the toxic effects of metal ions at trace levels towards living things, it is necessary to eliminate them from the environment. The essential trace metal ions monitoring from the environmental samples are paid significant attention in the present decade [156–161]. Although the detection of metals from the flame atomic absorption spectrometry (FAAS) is straightforward to operate, its level in the environmental samples is generally shallow than the FAAS detection limit. Also, it has a selectivity problem [162, 163 ]. The sample preparation and preconcentration step is essential during the metal determination [ 164–166]. The SUPRASs-based microextraction gained intensive attention by the researchers due to its simplicity and selective quantifications of metal ions from real samples. The SUPRASs consist of nanostructured liquids that make assemblies of amphiphiles dispersed into the aqueous media [167–169]. The SUPRASs were formed by the spontaneous reversed-phase aggregates of alkanols in tetrahydrofuran (THF)/aqueous solution via self-assembly processes. That can be applied for the extraction and preconcentration of inorganic substances. In the SUPRASs the aqueous cavities are produced by the surrounding aqueous phase by the polar group of alkanols with a chain of hydrocarbons dissolved in THF. Water/THF ratio in the balk amount of solution where the self-assemble of alkanols control can control the size of aqueous cavities the self-assembly and disperse of extraction solvent (decanol) in the solution by the THF. SUPRASs contain both hydrogen-bonding interactions and dispersion. Due to the nanostructured of SUPRASs that provide a suitable reaction media for the extraction and preconcentration of targeted ions [137]. The advantages of SUPRASs’ small amount of toxic solvents have been used for the extraction and sample preparation, and it is a speedy and easy method. Different researchers have reported other SUPRASs-based approaches for the extraction and preconcentration of metals from environmental samples. Table 2 represents the applications of SUPRAs for the extraction of metal ions. Rastegar et al. [170] proposed SUPRASs-based SM-DLLME method to extract lead from the actual samples. To remove SUPRASs using 1-decanol and THF as a solvent using a reverse-phase process into the aqueous solutions after micelles (nano-size) preparation. After that, the dithizone was used as a ligand for the complexing with the lead. After the practice of SUPRASs is applied to extract charge with the LOD value up to 0.4 μgL^−1^. The proposed SUPRASs-SM-DLLME method has successfully removed lead from the food and agriculture samples before FAAS analysis. Kashanak et al. [171] reported a new SUPRASs based D-μSPE method to extract copper from the food and water samples before the AAS analysis. The proposed methods obtained a good LOD value up to 0.2 ng mL^−1^. The developed method has been successfully applied for real food and water samples.

### 3.2. SUPRASs based extraction of different organic pollutants from different environment samples

SUPRASs are water-immiscible liquids that create molecular cavities that are dispersed in the continuous phase [188]. They are made from amphiphile solutions by the well-known self-assembly route occurring on two scales, atomic and molecular. The first amphiphilic molecule spontaneously forms 3D aggregate (aqueous and vesicles or reversed micelles). Then, the formation of nanostructures self-assemble in the enormous aggregates with large size distribution in the micro and nano-scale regimes by the action of an external stimulus such as type of electrolyte, temperature, pH solvent and separate it from the bulk solution through the mechanism that remains elusive. The liquid-liquid extraction phenomenon is named coacervation [189]. For years, SUPRASs have been prepared by the aqueous surfactant liquid micelles. They have been usually used to extract pollutants from the aqueous environment [190, 191 ]. The SUPRASs produced by vesicles and the reversed micelles of (alkyl carboxylic acids) have unique properties for the elimination process for various interactions points. They could be recognized with solute in the high concentration of amphiphiles [36]. Due to the novel properties of SUPRASs, they have been used to extract different organic compounds such as dyes, drugs, phenolic compounds, and some are listed in Table 3. María Jesús Dueñas-Mas et al. [192] have reported A new SUPRASs-based microextraction process for the extraction of BPA. Multitarget solvents have prepared SUPRASs to create self-assembled amphiphiles. The proposed method has been successfully applied to extract BPA from real samples. The proposed method obtained good LOD up to 6–22 ng g^−1^. Nail Altunay& Adil Elik [193] proposed SUPRASs VA-LPME to extract nitrite from the chicken meat products prior to spectroscopic determination. The proposed method obtained a good LOD value up to 0.035 ng mL^−1^.

### 3.3. Challenges and future visions

SUPRASs are greener and cheaper solvents that can be used as an efficient alternative to toxic organic solvents during the sample preparation and preconcentration of different targeted pollutants such as pesticides, metal ions, and organic contaminants from the environment, and complex samples. SUPRASs have outstanding properties due to the formation of different interactions, including hydrogen bonding, ionic bonding, and hydrophobic interactions. The physical and chemical properties of SUPRAS can be easily altered by varying the concentration and the type of amphiphiles. Furthermore, SUPRASs are greener and environmentally friendly, nonvolatile, and inflammable. These novel physical and chemical properties make them an efficient candidate for the alternative of organic solvents during the microextraction of targeted analytes. However, the usage of SUPRAS throughout the microextraction process is not free of challenges. During SUPRASs based microextraction, the THF is generally used as a dispersion solvent and to help the self-assembly of amphiphiles. THF poses a toxic environmental disquiet as the World Health Organisation categorized it in the 2 B class of carcinogens. Food, water, environmental, and complex biological samples are generally analyzed by modern analytical techniques such as GC-MS, HPLC, UPLC, LC/MS-MS, ICP, AAS. But these techniques are unable to examine the trace amount of pollutants from the complex samples due to this reason the SUPRASs based extraction has been recognized for the sample preparation and pretreatment before analysis. However, due to the low volatility of SUPRAS, the GC cannot analyze them. Due to the issue, the HPLC has been used to analyze targeted analytes after the SUPRASs based extraction. But the challenge during the HPLC analysis is that the most enriched SUPRASs have very high viscosity, making the analysis difficult.

To overcome this problem, researchers diluted (using suitable solvent) the enriched SUPRASs before chromatographic analysis. For instance, Deng et al. [139] used acetonitrile to dilute the enriched SUPRAS phase before LC-MS analysis. Scheel and Teixeira Tarley [173] diluted the enriched SUPRASs phase using methanol before HPLC-DAD analysis. The suitable organic solvents have been used during the dilution of enriched SUPRASs phased, but these organic solvents cause toxic environmental concerns. Although SUPRASs are considered greener and environmentally friendly alternatives to toxic organic solvents, their use still causes some environmental concerns. Thus, the research for the greener and environmentally friendly solvent should be incessant process among the scientists. The SUPRASs include possible greener alternatives such as sues of bio-solvents and liquid polymers and still; researchers explore the suitable alternative solvent for the microextraction of complex matrices. There are new amphiphiles that should be investigated, including alkyl sulfonates for the microextraction and preconcentration of samples from the complex matrix.

## 4. Conclusion

SUPRASs based microextraction is a green and environmentally friendly technique for sample preparation and preconcentration of targeted analytes from the complex food, water, environmental, and biological matrices. It contains the in-situ generation of SUPRASs by mixing an amphiphile with dispersion solvents in the aqueous media. The common amphiphile has been used during the SUPRASs based microextraction of toxic pollutants such as metal ions. Pesticides, toxic organic contaminants from the environmental matrices, are either long-chain carboxylic acids or long-chain alcohols, while the THF is used as a common dispersive solvent. The THF plays a vital role during the dispersion of amphiphiles and their self-assembly. The SUPRASs-based microextraction is generally considered a relatively cheaper greener, and it contains high relative enrichment factor and extraction efficiency. The centrifuge machine is usually used for the phase separation process during the SUPRASs based microextraction. The centrifugation step is time-consuming. The incorporation of ferrofluids bypasses this step during the microextraction of complex matrices using the SUPRASs. In ferrofluids, the phase separation process would be completed using the external magnetic field, and this novel addition in the SUPRASs opens a new door in the microextraction process. The unique combination of ferrofluids and SUPRAS minimizes the time consumption and extraction process.

## Figures and Tables

**Table 1:** The SUPRASs-based extraction of pesticides and herbicides from the different samples.

SUPRAS Components	Matrix	Target pesticide(s)	Instrumental analysis	Extraction recovery %	LOD (*μ* g L− 1)	Reference
Undecanol/Tetrahydrofuran and NaCl SUPRASs-Based Microextraction	Drinking and Environment Water	Fluorine-containing pesticides	LC-MS	81.3–105.9	0.42–0.84	[139]
Decanoic Acid/Tetrahydrofuran SUPRASs-Based Microextraction	Water and Rice	Herbicides: monuron, linuron and isoproturon	HPLC	80–99	30, 10, 30	[145]
Heptanol/Tetrahydrofuran SUPRASs-Based Microextraction	Water, Fruits and Vegetables	Carbaryl	LC-MS/MS	90–102	30	[138]
1-Decanol/Tetrahydrofuran and NaCl SUPRASs-Based Microextraction	Water from Natural and Artificial Sources	Carbendazim, Fipronil and Picoxystrobin	HPLC-DAD	93.5–110	0.023–0.045	[141]
Sodium Dodecyl Sulfate and Tetrabutylammonium Bromide SUPRASs-Based Microextraction	Water and Rice	Phenoxy acid Herbicides	HPLC	81–110, 81–108	0.001–0.002	[146]
Ferrofluid Mediated Calcined Layered Double Hydroxide@Tanic Acid-Based SUPRASs SUPRASs- Microextraction	Orange, Peach, Grape and Apple Juices	Organophosphates: Diazinon and metalaxyl	GC-FID	85–96.6	0.20, 0.80	[147]
Undecanol/Tetrahydrofuran SUPRASs-Based Microextraction	Orange Juice and Tap Water	Organophosphates: Chlorpyrifos, Diazinon and Phosalone	HPLC-UV	94	0.0050–0.0130	[140]
1-Decanol/Tetrahydrofuran and NaCl SUPRASs-Based Microextraction	Natural Waters	Herbicide: Diuron, Hexazinone, Ametryn and Tebuthiuron	HPLC-DAD	95–111	0.013–0.0145	[114]
Decanoic Acid/Tetrhydrofuran and NaCl SUPRASs-Based Microextraction	Rice, Cucumber and Tomatoes	Organophosphates: Ethion, Phosalone, Diazinon and Chlorpyrifos	HPLC-UV	102–178	0.005–0.0020	[143]
Heptanol/Tetrahydrofuran SUPRASs-Based Microextraction	Water, Gawafa, Bear, Eggplant and Tomatoes	Organophosphate: Malathion	UHPLC- MS	89–104	1.40	[128]
Decanoic Acid/Tetrabutylammonium Hydroxide/Water SUPRASs-Based Microextraction	Water, Apple, Pineapple and Peach	Organophosphates: Fenitrothion, Phosalone and Chlorpyrifos	HPLC-UV	92.2–110.5	0.10–0.35	[130]
Decanoic Acid/Magnetic Nanoparticles and Tetrabutylammonium Cation SUPRASs-Based Microextraction	Tap, River, and Spring Water	Triazine Herbicide	HPLC-UV	90.3–105	300–500	[144]
Environment-Friendly SUPRASs-Based Microextraction	Water and Rice Samples	Phenoxy acid Herbicides	HPLC	81–110–81–108	1–2	[146]
Undecanol- Based SUPRASs –Microextraction	Drinking and Environmental Water	Perfluorinated compounds and Fluorine-containing Pesticides	UPLC-Q-Orbitrap HRMS	97	0.125–0.250	[139]
Alkanol-Based SUPRASs- Microextraction	Fruit Juice and Tap Water Samples	Organophosphorus pesticides	HPLC	99.98	0.05–0.13	[140]
Nanostructured SUPRASs Microextraction	Soil	Sulfonylurea herbicides	HPLC-UV	89	0.5	[148]
SUPRASs	Water and Onion Samples	Dinitroaniline herbicides	HPLC	97.5–100	3.0–5.5	[145]
Non-Ionic Nonylphenol Ethoxylate Based SUPRASs	Real Water	Orthophosphate	UV-Vis	97.5–102.0	0.1	[149]
SUPRASs-Based Microextraction	Natural Waters	Herbicides	HPLC-DAD	95 – 111	0.13 – 1.45	[114]
SUPRASs As A Carrier For Ferrofluid	Water and Fruit Juice Samples	Organophosphorus pesticides (OPPs)	HPLC	92.2–110.5	0.1, 0.35	[130]
Magnetic Nanoparticle Assisted SUPRASs		Triazine herbicides	HPLC-UV/Vis	98	0.3, 0.5	[130]
tetrahydrofuran (THF) and decanoic acid (DeA)-based SUPRASs	Rice	Pesticide	HPLC-UV	99	0.05	[143]
decanoic acid-THF-based SUPRASs	Canal Water and Tape Water	Phenylurea Herbicides	HPLC	91.1–99	0.030	[145]
decanol in tetrahydrofuran/water- based SUPRASs	Beer Samples	Chiral triazole fungicide	LC-MS	100	0.24	[150]

**Table 2:** The SUPRASs based extraction of metal ions from the different real samples.

SUPRAS Components	Matrix	Target	Instrumental analysis	Extraction recovery %	LOD (*μ* g L− 1)	Reference
1-Decanol- Based SUPRASs- Microextraction	Food	Cobalt	MS-FAAS	100	1.89	[168]
Undecanol- Based SUPRASs	Water and Acid Digested Food	Aluminum	Spectroscopy	97	1.20	[172]
Reverse Micelles of 1-Decanol Based SUPRASs	Agricultural and Food Samples	Lead	FAAS	95	0.4	[170]
Ultrasound-Assisted Extraction Combined With SUPRASs -Based Microextraction	Medicinal Plant	Cadmium	TS-FF-AAS	95	0.1	[173]
SUPRASs -Based Microextraction	Food, Spices, and Water Samples	Copper	FAAS	95	1.4	[174]
1- Decanol Based SUPRASs	Environmental Samples	Copper	FAAS	101	0.52	[175]
Vortex-Assisted SUPRASs	Environmental and Biological	Mercury	UV–Vis	96	0.30	[176]
1-decanol/THF- based SUPRASs Microextraction	Environmental Samples	Thorium	UV–Vis	98	0.40	[177]
SUPRASs	Environmental Samples	Mercury	AAS	95	0.561	[178]
Nano-Structured SUPRASs Microextraction	Hafnium	Zirconium	ICP-AES	=	0.1	[179]
Metal-Organic Framework Based Micro Solid Phase Extraction Coupled With 2 SUPRASs	Water and Food	Copper	AAS	95	0.02	[171]
THF and 1-decanol-based SUPRASs	Water	Cobalt	FAAS	99	1.29	[124]
Undecanol–THF-based SUPRASs	Water and Hair	Aluminum	UV-Vis	102	0.47	[167]
DeA-THF–water- based SUPRASs	Food and Water	Lead	GFAAS	100	0.027	[180]
1-decanol and THF-based SUPRASs	Environmental Samples	Gold	FAAS	97	1.5	[131]
SUPRASs	Water and Soil Samples	Uranium	UV-Vis	96	0.31	[181]
1-decanol/THF- based SUPRASs	Water and Hair Samples	Cobalt	FAAS	95	0.11	[168]
SUPRASs	Water	Copper	FAAS	96	0.46	[182]
Nonanoic acid, THF and water – based SUPRASs	Water	Copper and lead	FAAS	91.2–102.1	0.29	[132]
Decanoic acid and quaternary ammonium- based SUPRASs	Rice Samples	Cadmium	FAAS	93–107	0.09	[183]
1-decanol-THF based SUPRASs	Vegetables	Manganese and zinc	FAAS	98	0.06	[184]
THF- Decanoic acids- Based SUPRASs	Water Samples	Chromium	UV-Vis	98	0.79	[185]
1-decanol/THF-based SUPRASs	Water	Mercury	UV–Vis	103	2.6	[186]
1-decanol/THF-based SUPRASs	Water and Road Dust Samples	Palladium	FAAS	96–104	0.63	[187]

**Table 3:** The SUPRASs-based microextraction of different organic pollutants.

SUPRAS Components	Matrix	Target pesticide(s)	Instrumental analysis	Extraction recovery %	LOD (*μ* g L− 1)	Reference
Anion SUPRASs -Based Microextraction	Vegetable	Quats	HPLC	75.0–106.7	1.5, 2.8	[194]
Decanoic Acid- Based SUPRAS- Microextraction	Water	Malachite green	UV–vis	106	16.3	[129]
Hexanol - Based SUPRAS-Microextraction	Dust From Public Environments	Bisphenol A	LC-MS/MS	50–90	0.6–0.22	[192]
Micelles Of Decanoic Acid- Based SUPRASs- Microextraction	Raw Wheat	Ochratoxin A	LC-FL	84–95	5.0	[195]
Micelles Of Decanoic Acid- Based SUPRASs –Microextraction	Chilli, Foodstuffs	Sudan dyes	LC	86–108	6.5	[121]
Decanoic Acid- Based- SUPRASs-Based Microextraction	Sediment	Anticonvulsant and Nonsteroidal Anti-Inflammatory Drugs	UHPLC-UV	81.0–106	0.42	[196]
Decanoic Acid- Based- SUPRASs	Artificial Sweat S and Water	Sudan Orange G	UV-Vis	100	3.4	[197]
Reverse Micelle-Based SUPRASs	Water Samples	Triazines (Cyanazine, Simazine, Prometon and Propazine	HPLC-UV	=	0.3, 0.5	[127]
Vortex-Assisted SUPRASs -Based Liquid Phase Microextraction (Va-Supras-Lpme)	Processed Meat and Chicken Products	Nitrite in	UV–VIS	95.0–102.5	0.0035	[193]
Aerosol Into-SUPRASs-Based Microextraction	Respirable Dust	Crystalline silica	UV–VIS	100.8	2.85	[149]
Nano-Structured SUPRASs Based On Propanol/Gemini Surfactant -Based Microextraction	Cosmetics, Beverages, and Water Samples	Parabens	HPLC	92.0–108.3	0.7	[115]
1-Decanol-Based SUPRASs -Based Microextraction	Water and Food Samples.	Manganese ethylene-bisdithiocarbamate	UV-Vis	98	2.22	[198]
Nano-Sized Inverted Hexagonal Aggregates of 1-Octanol-Based SUPRASs	Unburned Single-Base Propellants	Diphenylamine and its mono-nitrated derivatives	UV–Vis (DAD)	82.6–98.7	0.05–0.12	[199]
Oleic Acidcoated Magnetic Particles and The SUPRASs	Biological Samples	Levofloxacin (LEVO)	Varian Cary Eclipse spectrofluorometer	94.0–106.0	0.02	[200]
Vortex-Assisted SUPRASs Liquid–Liquid Microextraction	Environmental Water	Nitroaniline isomers	HPLC	104.0	0.3	[201]
Magnetic Dispersive Micro Solid-Phase Extraction And SUPRASs-Based Microextraction	Complicated Matrices	Cholesterol-lowering drugs	HPLC	86	0.03	[202]
Reverse Micelles of Decanoic Acid SUPRASs	Meat	Sulfonamides	EC	98–109	12	[203]
Pentanol Based SUPRASs	Rats With Type 1 Diabetes	Aucubin	UPLC-MS/MS	88.6	0. 10	[204]
Decanoic Acid (Dea) And Tetrabutylammonium Decanoate (Bu4nde) Based SUPRASs	Fruits and Vegetables	Benzimidazolic fungicides	LC-FID	93–102	14	[116]
Phase-Transfer-Catalyst-Assisted Saponification And SUPRASs	Edible Oils	Benzo[a]pyrene	HPLC-FID	102	0.06	[205]
Vortex-Assisted SUPRASs Microextraction	Liquid Foods and Their Packaging Materials	Bisphenol-A, 2,4-dichlorophenol, bisphenol-AF and tetrabromobisphenol-A	HPLC	91–105.1	0.014–0.032	[206]
Vesicular Based-SUPRASs Microextraction	Fruit Samples	Diphenylamine	HPLC-UV	90–101	3	[207]
Vesic-Ular SUPRASs-Based Microextraction	Food	Polycyclic aromatic hydrocarbons (PAH4)	LC-FID	92–103	0.3–0.7	[134]
SUPRASs (SUPRAS) Made Up Of Decanoic Acid (Dea)	Salmonids	Astaxanthin and canthaxanthin	LC-UV/Vis	94–106	0.4	[208]
Upramolecular Solvent Microextraction	Water, Vegetables, and Fruit	Vanadium	AAS	95.0–104.8	0.12	[209]
Vesicular Aggregate-Based SUPRASs	Water	Halogenated anilines	HPLC	90.4–107.4	0.5–1.0	[210]
Ultrasonic-Assisted Restricted Access SUPRASs-Based Liquid Phase Microextraction	Herbal Tea, Turmeric Powder, Syrup, and Drug	Curcumin	UV-VIS	100	17.5	[211]
SUPRASs Microextraction	Water, Fruits and Vegetable	Carbaryl	UPLC-MS	90–102	0.3	[138]
SUPRASs Based Microextraction	In Environmental Water	Phenols	HPLC	82–105	1–4	[212]
Alkanol-Based Nano Structured SUPRASs	Biological Samples	Antidepressant drugs	GC-MS	91–102	0.003	[213]
SUPRASs-Based Microextraction	Distilled Water and Plasma Samples	Loratadine	HPLC	92	0.03–0.04	[214]
SUPRASs-Based Microextraction	Soils	Mecoprop and dichlorprop	LC–MS/MS	93–104	0.01	[215]
Hexafluoroisopropanol/Brij-35 Based SUPRASs	Water Samples, Pharmaceuticals and Personal Care Products	Parabens	HPLC	101	0.042 to 0.167	[216]
Hexafluoroisopropanol-Alkyl Carboxylic Acid High-Density SUPRASs	Human Urine	Steroid sex hormones	LC-MS	84–106	0.01–0.10	[216]
SUPRASs	Environmental Water	Nitrophenols	HPLC-UV/Vis	94	0.26, 0.58	[217]
Ultrasonic Assisted SUPRASs	Water and Beverage	Erythrosine	UV/Vis	95	0.06	[218]
SUPRASs based Microextraction	Solid Cream Samples	Paraben	LC-UV/Vis	86–102	0.03–0.04	[219]
Ultrasonic-Assisted SUPRASs	Biological Samples	Antifungal drugs	HPLC	96	0.08–1.3	[220]
Ultrasonic-Assisted Restricted Access SUPRASs	Food	Quercetin	UV/Vis	87–104	2.98	[221]
Nano-Structured Gemini-Based SUPRASs	Water and Soil Samples	Cyhalothrin and Fenvalerate	HPLC	101.2–108.8	0.2	[222]
SUPRASs Microextraction	Water	Ethinyl estradiol	HPLC	93	0.1	[223]
Decanoic acid THF-based SUPRASs	Foodstuff Samples	Food dyes	HPLC-UV	85.5–108	0.05–0.1	[224]
1-octanol-THF-based SUPRASs	Food and Environmental Samples	Selenium	FAAS	98	0.1	[225]
Quick SUPRASs	Soy Foods	Isoflavones	UHPLC	90.3–105	0.7	[226]
Quick SUPRASs	Food	Curcuminoid	LC- PDA	85	2.9	[227]
Property SUPRASs	Sediment	Endocrine disruptors	LC/MS-MS	93–104	0.064	[117]
SUPRASs Based Magnetic Solvent	Human Serum	Non-steroidal anti-inflammatory drugs	LC/MS-MS	86.8–125.1	0.83–3.16	[228]
1-decanol/THF/water-based SUPRASs	Artificial Occurring Water Bodies	Carbendazim, Fipronil and Picoxystrobin	HPLC-DAD	84.47, 83	0.78–1.50	[141]
1-decanol/THF SUPRASs	Tap Water, Lipstick, Rouge, and Nail Polish	Rhodamine B	UV-Vis	95	0.49	[229]
DeA/THF-based SUPRASs	Whole Blood Samples	Levonorgestrel and Megestrol	HPLC/UV	90–98	1–2	[230]
SUPRASs	Apple Peels	Polycyclic aromatic hydrocarbons	HPLC	99	0.34	[231]
Decanoic acid/THF-based SUPRASs	Water Sample	Uranyl ion	UV/vis	104	0.002	[218]
SUPRASs	Environmental	Malathion	UPLC/MS-MS	90	1.4	[232]
SUPRASs	Environmental	Sudan blue II	UV-Vis	100	2.16	[233]
SUPRASs	Water, Fruit Juice, Plasma and Urine	Benzodiazepines	HPLC-DAD	90.0–98.8	.5–0.7	[127]
SUPRASs	Indoor Dust From Houses	Aryl-phosphate flame retardants	LC	120	0.5–10	[192]
SUPRASs	Biological	Warfarin	HPLC	96	14.5	[234]
SUPRASs	Water Samples	Glucocorticoids	HPLC	103	2.4	[235]
SUPRASss	Biological Fluids	Amine and monoterpenoid	HPLC-UV	93	0.06	[236]
SUPRASss	Environmental Water	Inorganic species	AAS	103	0.55	[237]
SUPRASss	Food Samples	Orange II	UV/Vis	91	0.35	[238]
SUPRASss	Water	Carbaryl	HPLC	96–105	0.3–1.0	[239]
Volatile SUPRASs	Urine	Bisphenol A	LC-(ESI)MS/MS	96–107	0.025	[28]
SUPRASs	Human Plasma and Saliva	Methadone	GC–MS	92	0.5–1.2	[240]
Novel Ultrasonically Enhanced SUPRASs	Water and Cosmetics	Phthalates	HPLC-UV	91.0–108	0.10–0.70	[241]
Ultrasound-Assisted SUPRASs	Biological, Environmental, and Food Samples	Inorganic arsenic	GFAAS	95	0.002	[242]
Ultrasound-Assisted SUPRASs	Environmental Water Samples	Chlorophenols	HPLC	83.0–89.3	0.0015–0.0020	[243]
Vesicular SUPRASs	Milk, Egg and Honey Samples	Tetracyclines	HPLC	110	0.7–3.4	[119]
Vortex Assisted-SUPRASs	Environmental Water Samples	Triclosan	UV/Vis		0.28	[244]
Vortex-Assisted SUPRASs	Water	Inorganic arsenic	UV/Vis	105	0.4	[245]
